# Object exploration facilitates 4-month-olds’ mental rotation performance

**DOI:** 10.1371/journal.pone.0200468

**Published:** 2018-08-09

**Authors:** Lauren K. Slone, David S. Moore, Scott P. Johnson

**Affiliations:** 1 Department of Psychological and Brain Sciences, Indiana University, Bloomington IN, United States of America; 2 Psychology Field Group, Pitzer College and Claremont Graduate University, Claremont CA, United States of America; 3 Department of Psychology, University of California, Los Angeles, Los Angeles CA, United States of America; Medical Photonics Research Center, Hamamatsu University School of Medicine, JAPAN

## Abstract

How do infants learn to mentally rotate objects, to imagine them rotating through different viewpoints? One possibility is that development of infants’ mental rotation (MR) is facilitated by visual and manual experience with complex objects. To evaluate this possibility, eighty 4-month-olds (40 females, 40 males) participated in an object exploration task with Velcro “sticky mittens” that allow infants too young to grasp objects themselves to nonetheless explore those objects manually as well as visually. These eighty infants also participated in a visual habituation task designed to test MR. Half the infants (Mittens First group) explored the object prior to the MR task, and the other half afterwards (Mittens Second group), to examine the role of immediate prior object experience on MR performance. We compared performance of male and female infants, but found little evidence for sex differences. However, we found an important effect of object exploration: The infants in the Mittens First group who exhibited the highest levels of spontaneous object engagement showed the strongest evidence of MR, but there were no consistent correlations between these measures for infants in the Mittens Second group. These findings suggest an important contribution from object experience to development of MR.

## Introduction

Mental rotation (MR) refers to the ability to rotate representations of objects in one’s mind. This ability has been studied extensively since the 1970s [1, 2] because of its theoretical importance to cognitive scientists [3] as well as its practical importance for a variety of everyday tasks (e.g., perspective taking and navigation [2], recognizing individual letters of the alphabet [4,5]). MR is also of broad interest to cognitive scientists because it is one of the few perceptual-cognitive abilities for which robust sex differences have been found across cultures [6,7] and across the lifespan [8–14]. The means by which MR develops in childhood is also of great interest, owing to its relevance to spatial, motor, and object perception skills more broadly [15]. In the current study, we test MR in 4-month-old infants to evaluate the possibility that visual and manual experience with objects early in life facilitates MR performance.

MR was first documented in adults by Shepard and Metzler [16]. Participants were presented with pairs of two-dimensional (2D) drawings representing three-dimensional (3D) objects and asked to determine whether or not the depicted objects could be rotated into alignment. Shepard and Metzler found that the angular difference between the orientations of the depicted objects was linearly related to the time it took adults to correctly identify pairs of identical objects. The interpretation was that participants maintained analog mental representations of the objects such that it took longer to imagine an object rotating into alignment with its comparison object as the difference in orientation between the objects increased.

Despite a large literature on MR in adults and a growing body of research on MR in infants and children, little is known about the factors underlying the development of this perceptual-cognitive skill. Given that MR performance is related to mathematics ability (e.g., [17, 18] and is predictive of later careers in STEM (science, technology, engineering, and mathematics) disciplines (e.g., [19, 20]), understanding its development is imperative.

### MR across the lifespan

In developmental research, MR tasks similar to those of Shepard and Metzler [16] have been successfully adapted for children as young as 4–5 years of age (e.g., [21–25]). Many MR studies with children and adults report that males perform faster and more accurately than females (e.g., [8, 10–14, 26, 27]). Moreover, an early meta-analytic study reported a large estimate of effect size (*d* = 0.73) for the sex difference in MR and suggested that this sex difference emerges at least by childhood and remains relatively constant through adulthood [12]. Nevertheless, findings of sex differences in children’s mental rotation are inconsistent (see [28] for review). Notably, a meta-analysis [14] that included the studies analyzed by Linn and Peterson [12] as well as 49 additional effect sizes, documented an increase in effect size with age, from 0.33 in children (under 13 years), to 0.45 in adolescents (13–18 years), to 0.66 in adults (over 18 years), suggesting that early in life, the sex difference in MR may not be as robust as was once thought (see [15] for discussion).

**Fig 1:**
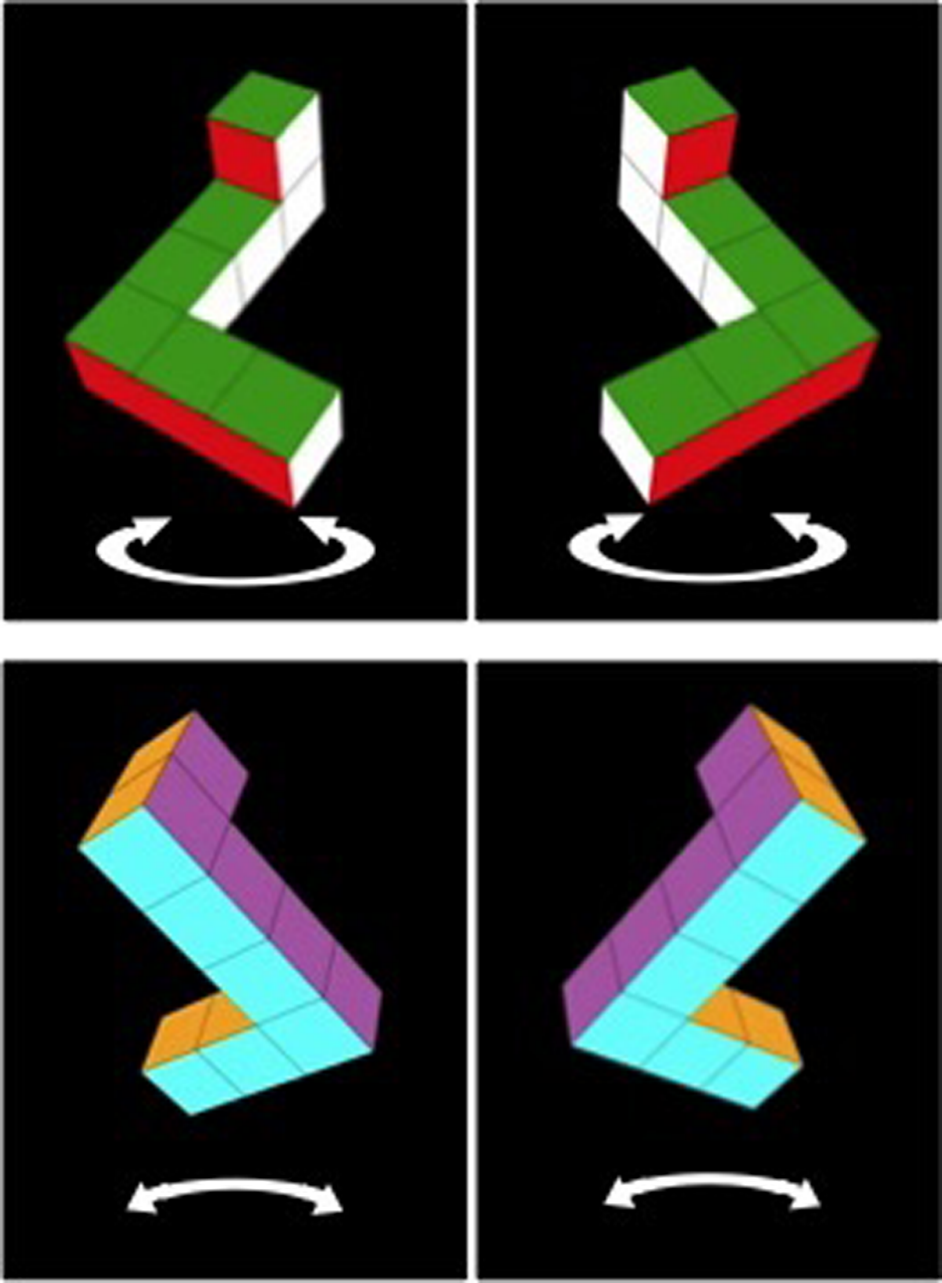
Stimuli. Top row: Still images of rotating objects shown during habituation. Infants were habituated to one of the two objects. Bottom row: Objects shown after habituation. Infants viewed both objects on alternating trials. See text for details.

Experiments investigating MR in infants and toddlers require methods such as habituation and violation of expectation that are quite different than those used with children and adults (see [29] for a review). Several studies have reported a male advantage in MR in infants as young as 3–5 months of age [30–36], consistent with the male advantage found in adults. Moore and Johnson [32], for instance, designed a visual habituation task to assess infants’ MR of 3D objects similar to those used by Shepard and Metzler [16]. Infants were habituated to a video representation of a 3D object rotating through a 240° angle around the vertical axis (see Fig 1). Following habituation, infants were presented with a series of alternating test trials in which they were shown either the original, familiar object, now rotating through the previously unseen 120° angle, or a mirror-image version of that stimulus, rotating through the same 120° angle. Both test stimuli therefore presented novel views of the objects to the infant. Moore and Johnson [32] found that male, but not female, 5-month-olds looked reliably longer at the mirror-image test stimulus, suggesting that only male infants recognized (and were therefore less interested in) the original habituation object, even when it was seen from the novel, rotated perspective.

Sex differences in infants’ MR performance are not always clear, however. Beginning in the late 1990s and continuing into the present, several studies on MR in infancy [29, 37–42] reported no evidence for sex differences in MR between 4 and 16 months. These conflicting results have been hypothesized to reflect differences in the dimensionality of the stimuli presented [29, 38]. Specifically, some studies employing 2D stimuli [31, 34, 35] and computer-generated 2D representations of 3D stimuli [30, 32, 33, 36] have shown sex differences, whereas studies employing live 3D presentations or videos thereof [29, 37, 38] have not found sex differences. However, the specific mechanisms underlying the proposed dimensionality distinction have not been described, and this explanation is inconsistent with research with children and adults that finds no differences in reaction time between MR of 2D and 3D objects [2] and meta-analyses reporting larger sex differences for MR of 3D compared to 2D objects [12, 14] (see [35] for a discussion). It remains unclear whether and how dimensionality differences might relate to the inconsistent findings of sex differences in MR among infants. Nevertheless, evidence for early sex differences remains mixed.

The present study was designed to examine a theoretically motivated factor that may support the development of MR in infancy: object exploration. Four-month-olds wore Velcro-covered mittens, allowing them to “grasp” and explore objects. We hypothesized that individual variation in visual and manual exploration of objects might relate to differential MR ability (cf. [42]).

### Object exploration and MR

Object exploration undergoes major development in the first 6 postnatal months. Infants engage in far more exploration over time [43]. For instance, 2- and 3-month-olds initially explore objects orally, and begin to explore objects visually by 4–5 months. Object exploration also becomes increasingly multimodal [43]. Around 5–6 months of age, infants begin sitting independently, freeing their hands from having to support themselves and allowing infants to coordinate their visual and manual exploration of objects [44, 45].

Visual object exploration may contribute to the development of MR ability in infancy. Mash, Arterberry, and Bornstein [46] familiarized 5-month-olds to either a single view or multiple views of a novel object and subsequently tested infants’ recognition of that same object in different orientations. Only infants who had observed the object from multiple perspectives provided evidence of recognizing the object in a novel orientation, suggesting the importance of visual object exploration for MR ability.

Moreover, the ability to coordinate visual and manual object exploration, and therefore to generate multiple object views for oneself, appears to contribute to development of object representations in infancy. Needham [47] found that 3- and 4-month-olds who engaged in greater proportions of coordinated visual-manual object exploration showed enhanced object segregation skills in a violation-of-expectation paradigm. Additionally, Soska and colleagues [45] conducted a study of infants’ 3D object completion—the ability to infer the solid 3D shape of objects seen from a limited vantage point—and found that coordinated visual-manual object exploration was the strongest predictor of 4- to 7-month-olds’ performance. Thus, we hypothesized that the development of visual object exploratory skill, particularly when coordinated with manual exploration, may facilitate MR ability by giving infants experience with multiple views of objects over time.

Although infants observe objects in their environment from multiple views prior to the ability to produce these views themselves by manipulating objects, a growing body of research and theory suggests that infants’ *self-produced* actions affect their cognitive and perceptual development in ways that simply observing the causes and effects of others’ actions do not. For instance, Sommerville, Woodward, and Needham [48] found that 3-month-olds whose grasping of objects was facilitated via Velcro mittens later interpreted adults’ reaches for the same objects as goal-directed; infants who observed another person grasp the object rather than grasping it themselves did not. The idea that there is a close linkage between motor and perceptual systems has a long history, going back to early theories of cognitive development [49–51]. The idea continues to play a central role in more recent theories of cognitive development (e.g., [52]) and has gained new popularity in the field of embodied cognition [53, 54].

Interestingly, recent research suggests that self-produced action may be especially important for the development of spatial abilities [55], and of MR in particular. Correlational analyses suggest that 9-month-olds’ crawling experience is associated with MR performance [42] and at least two studies suggest a relation between manual exploration and MR. Frick and Wang [38] demonstrated that 13- and 14-month-old infants were able to mentally track the orientation of an object on a turntable only after hands-on experience with the turntable carrying another object, and Möhring and Frick [29] found that 6-month-old infants showed evidence of successfully mentally rotating an object only if they had the opportunity to visually and manually explore the test object prior to the experiment. Notably, however, 6-month-olds given the opportunity to manually explore the object prior to test did so while the object was held and rotated by an experimenter, such that the visual information provided to the infant was produced primarily by the experimenter’s, rather than by the infant’s, manual actions.

Such findings demonstrate a relation between object exploration and spatial ability, yet the role that infants’ observation of their *own* object manipulation plays in the development of MR remains unclear. The present study investigated this question by facilitating object exploration in 4-month-old infants; as noted previously, infants at this age are generally not able to grasp objects, thus precluding opportunities for manual or coordinated visual-manual object exploration. We fitted infants with Velcro “sticky” mittens, which have been shown to facilitate object exploration in pre-reaching infants (e.g., [55, 56]). We hypothesized that facilitating manual engagement via Velcro mittens would promote infants’ creation and observation of multiple views of objects over time, resulting in more elaborated mental representations and enhanced MR performance.

### The current study and hypotheses

The goal of the present experiment was to advance our knowledge of MR development in at least two ways. First, although studies have demonstrated a relation between visual-manual exploration and MR, these studies involved infants’ visual exploration following manual actions produced either by an experimenter [29] or by infants already capable of relatively skilled manual object exploration [38]. We designed an experiment to examine whether infants’ own manual actions on objects, and visual exploration of those objects, play a causal role in the development of infants’ spatial skills. We observed 4-month-old infants, not capable of skilled reaching, grasping, or coordinated visual-manual exploration of objects, and facilitated such experiences using Velcro mittens. Infants were randomly assigned to engage in the object exploration task either prior to or following our assessment of MR (i.e., “Mittens First” vs. “Mittens Second” conditions). This manipulation of task order was based on previous studies (e.g., [48]), and allowed us to assess whether self-generated experience exploring objects directly prior to viewing the visual habituation object enhanced infants’ ability to discriminate that object from its mirror-image test object (i.e., MR ability). We operationalized MR performance in terms of a posthabituation preference for the novel, mirror-image test stimulus, an indication that infants recognized that the object seen in the “familiar-object” test trials was the same object seen during habituation, but from a different viewpoint [32]. In contrast, the mirror-image object seen in the “novel-object” test trials was even more novel than the habituation object seen from a new perspective, which explains why the mirror-image test stimulus might recruit attention more than the familiar test stimulus, even though neither test stimulus had previously been seen.

Second, in contrast with previous studies, the present study was designed to investigate group *and* individual differences on a MR task as well as in self-produced visual and manual object exploration behaviors. If mittens-facilitated object exploration promotes MR performance, we should find a relation between MR performance and object exploration only in the condition in which exploration *precedes* the MR task. If MR is related to exploration that occurs both before *and after* the MR test, this suggests that it is not exploration experience *prior to* an MR task that is critical for facilitating MR, but rather that infants already capable of MR also tend to explore objects more.

Our design allowed us to test several important predictions about MR performance in infancy and the means by which it may be facilitated. First, we predicted that males and females may exhibit different patterns of performance, given previous reports of a male advantage in young infants’ MR [30–36]. Second, we predicted that infants in the Mittens-First condition may outperform infants in the Mittens-Second condition, given previous reports that sticky mittens training has positive effects on complex object processing [57], object-directed activity [56]. and perception of object-oriented behaviors in others [48]. Third, we predicted that effects of mittens experience may be realized more fully in individual infants who are more spontaneously engaged with the objects, given previous reports that motor skills, self-produced multimodal object exploration, and object knowledge emerge in tandem across infancy [43, 45, 47]. Comparing individuals’ responses in the Mittens First and Mittens Second conditions allowed us to examine whether such effects are general, in which case the two conditions would not be expected to differ, or whether the effects might be specific to the Mittens First group, which might indicate that self-produced object exploration has a training effect.

## Materials and methods

### Participants

Participants were 104 healthy, full-term 4-month-old infants, recruited in a large metropolitan area of the southwestern United States by letter and telephone from county records. Infants were included in the final sample if they completed the object exploration phase and at least the first two (of three) test blocks of the MR task; 24 infants were excluded due to fussiness or disinterest (*n* = 12), technical failure (*n* = 9), inability to wear mittens (*n* = 2), or parental interference (*n* = 1). Eighty infants (*M* age = 138 days, *SD* = 10 days) composed the final sample, with 20 males and 20 females in each of two conditions, Mittens First or Mittens Second. Power analyses based on the effect size (*d* = 0.61) for male infants’ MR reported in Moore and Johnson (2008) indicated that a sample size of 20 infants per group would yield power = 0.74. All infants were given a small gift (a toy or baby t-shirt) for participating. The study’s protocol was approved by the UCLA North General Institutional Review Board. Participants were treated in accordance with University IRB #10–000619, “Brain Mechanisms of Visual Development.” A parent provided written consent for each infant participant.

### Procedures and stimuli

There were two parts to the study: visual habituation in a MR task, and object exploration. Data collection was completed during a single visit to the lab. The dependent variable of interest was MR performance, operationalized as novelty preference following visual habituation to stimuli designed specifically for assessing MR performance (see Fig 1). Task order was manipulated as a between-subjects variable: Infants were randomly assigned to participate in the object exploration task either prior to the MR task (Mittens First condition), or after the MR task (Mittens Second condition).

#### MR performance in a visual habituation task

Infants participated in a visual habituation procedure similar to that used by Moore and Johnson [38]. Stimuli consisted of 2D depictions of unfamiliar 3D objects representing simplified Shepard-Metzler objects [16]. Objects were constructed of seven colored cubes that were attached to form an object with three segments: a top two-cube segment, a bottom three-cube segment, and a middle two-cube segment that was aligned at 90° angles with both the first cube of the top and bottom segments (see Fig 1). Objects were presented against a black background and each of the six faces of the object was a different color (red, green, white, blue, yellow, and purple).

Infants sat in a caregiver’s lap in a darkened room, 60 cm from a 53-cm screen on which all stimuli were presented. Caretakers were instructed not to interact with the infant or attend to the monitor. A Macintosh running Eprime software presented stimuli and collected looking time data. The computer calculated the habituation criterion online for each infant, and changed displays after the habituation criterion was met. A trained observer, not visible to the infant and blind to the stimulus shown, observed the infant via a video feed from a camera placed directly below the monitor; this observer coded looking time behavior online by pressing and releasing a preset keyboard key. Each habituation and test stimulus was presented until the infant visually fixated the stimulus for a total of 90 s, or until the infant looked away from the monitor for over 2 consecutive s (whichever came first). Prior to each trial, one of several “attention-getters” (small animated pictures of toys accompanied by different sounds) was shown to attract infants’ gaze to the center of the screen, whereupon the next trial was started immediately.

Two habituation videos and two test videos were used. Habituation videos presented either an object arbitrarily designated the “right-handed” R-object, or its mirror image, designated the “left-handed” L-object (see Fig 1, top). Each habituation object rotated back and forth at 30° per second through 240° around the vertical axis and subtended a maximum visual angle of 15.3° (height) and 11.4° (width). Infants were randomly assigned to see a series of identical habituation trials presenting either the R- or L-object. The habituation trials continued until looking time to a block of four trials reduced below 50% of the infant’s looking time to the first four trials. Thus, infants saw the habituation display for a minimum of 5 trials. If an infant did not meet the habituation criterion after 12 trials, the experimenter presented the test displays anyway.

Following habituation, infants were presented with six test trials alternating between the R- and L-test objects, each rotating through the previously unseen 120° angle (see Fig 1, bottom). Half of these test trials were “familiar-object” test trials, in which the infants saw the same object seen during habituation, but from a new viewpoint; the other half were “novel-object” test trials, in which the infants saw the other (mirror-image) object rotating through the same new viewpoint. Which test object each infant viewed first was randomized. A second judge who was blind to the hypotheses and blind to which trial type was presented recoded the looking times of 30% of the infants from digital recordings of the sessions. The Pearson correlation between the two coders’ scores was .80 and the mean difference between the scores was 0.35 s, *p* = 0.297.

#### Object exploration

**Fig 2:**
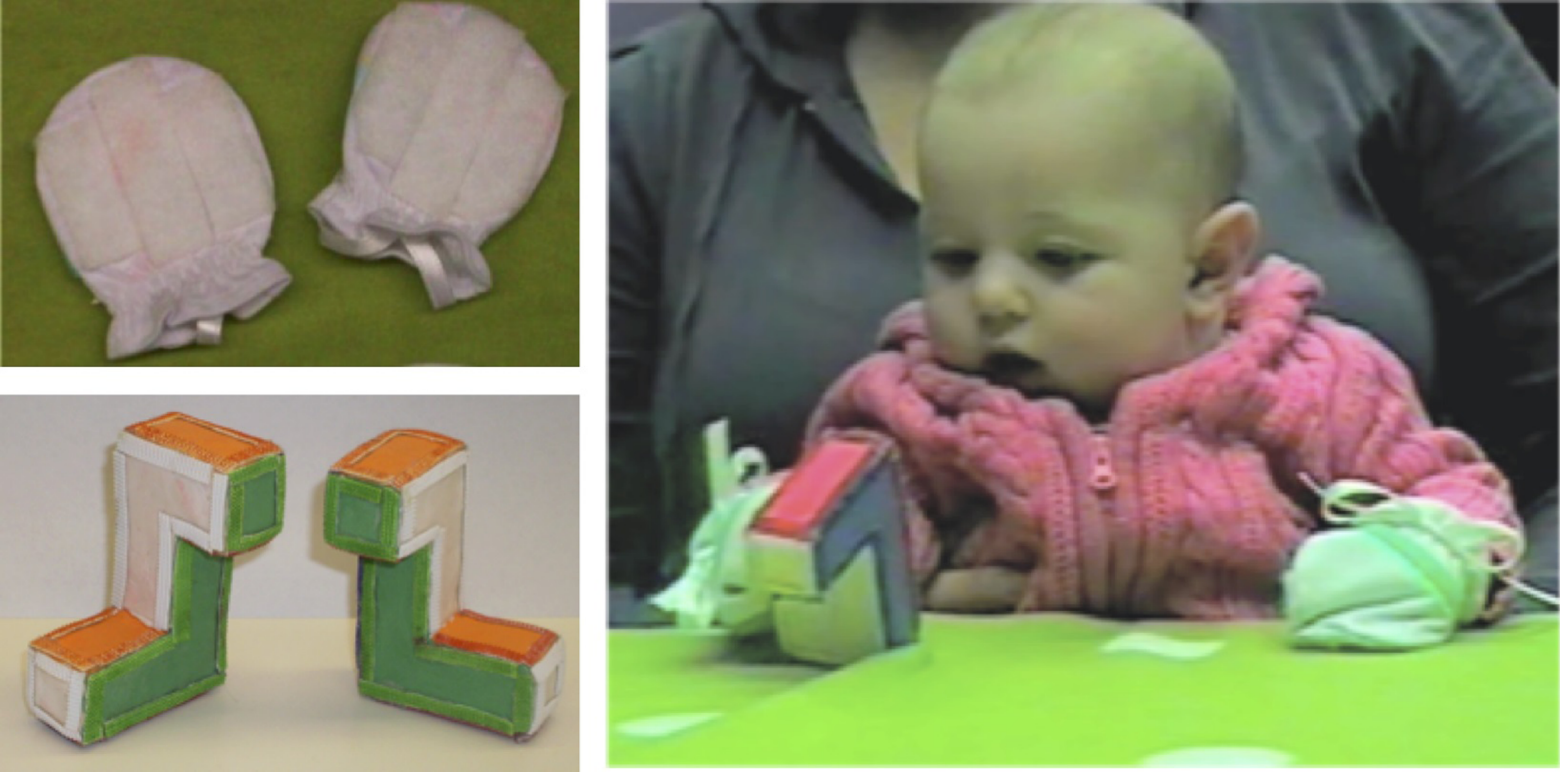
Object exploration task. Left, top: Velcro mittens worn by infants in the object exploration task. Left, bottom: The two objects presented to infants during the object exploration task. Right: An infant participating in the object exploration task. (The infant’s parent provided written informed consent to publish this image).

Infants sat on a caregiver’s lap, supported at the torso, directly in front of a table at chest level, and wore a pair of white mittens covered in “loop” Velcro (see Fig 2). The mittens were fashioned after those used by Needham and colleagues [56] and allowed infants to make contact with and pick up “hook” Velcro-covered objects by swiping or batting at them. Both objects were 7–8 cm long and were made of colored foam covered in hook Velcro. The experimenter offered infants two objects, one at a time in alternation for two trials each. The objects were mirror images of one another and made to be identical in color and proportion to the R- and L-habituation objects. Which object was presented first was randomly assigned and counterbalanced across infants.

Each of the four 60-s object exploration trials began with the experimenter presenting the object at midline. Objects were presented to infants from the same orientation on all trials. All trials were recorded with a digital video camera situated to provide a head-on view of infants’ activity (see Fig 2, right). The experimenter responded in a standard fashion when one of the following four types of events occurred, none of which occurred frequently across the participant population. If an infant did not make contact with the object within 10 s of its presentation, the experimenter brought the infant’s closest mitten into contact with the object. If both of an infant’s mittens became attached to the Velcro object and the infant could not separate either mitten from the object, the experimenter removed one mitten from contact with the object after 3 s. If an infant’s mittens were pulled off, the object was taken out of sight while the mittens were replaced, and then the trial resumed. If an infant broke visual and manual contact with the object for greater than 5 s, the experimenter tapped on the table next to the object and said “Look, *infant’s name*, look” to attract attention back to the object. After 60 s, the experimenter detached the object from the infant’s mittens and offered the next object.

Digital recordings of infants’ object exploration were analyzed using MacSHAPA (www.openshapa.org; [58]) for coding of frequencies and durations of infants’ actions (see [45]). Recordings were coded for durations of looking only (looking at an object without contacting it), touching only (manual contact without looking), and looking while touching. A second coder who was blind to the hypotheses recoded the behavior of 25% of the infants. The Pearson correlation between the two coders was > .90 for all three measures.

#### Design and analysis

As noted previously, we operationalized MR performance in terms of a posthabituation preference for the novel, mirror-image test stimulus. *Novelty Preference* was computed as average looking time toward the novel test object minus average looking time toward the familiar test object. Object exploration was operationalized as the duration of looking and touching the object. There were two key measures used in our analyses: coordinated *Visual-Manual Exploration*, defined as the duration in seconds that infants looked at the object when it was in contact with the mitten (cf. [45]), and *Total Engagement*, defined as the sum of Visual-Manual Exploration, looking at the object without touching, and touching the object without looking. We first report group differences in MR performance and object exploration, and then analyze for relations between these measures in terms of individual differences.

## Results

### MR performance

Infants were presented with three blocks of test trials, each block consisting of two trials, one trial presenting the familiar habituation object (seen from a new viewpoint) and the other trial presenting the novel, mirror-image object. Looking time distributions across the six cells (three familiar and three novel) were positively skewed, ranging from 1.93–5.88 (*M* = 2.90), violating assumptions underlying parametric analysis. We therefore winsorized scores by replacing the highest and lowest 20 looking times across the entire sample (8% of scores) with the next highest and lowest looking times, respectively. This reduced skew in looking time distributions to a range of 1.15–1.69 (M = 1.35). (Measures of Visual-Manual Exploration and Total Engagement were not overly skewed, .37 and -1.07, respectively, and so those scores were not winsorized.) Six infants failed to complete the three blocks of test trials, and for these infants we averaged over the other two blocks to compute novelty preference.

**Fig 3:**
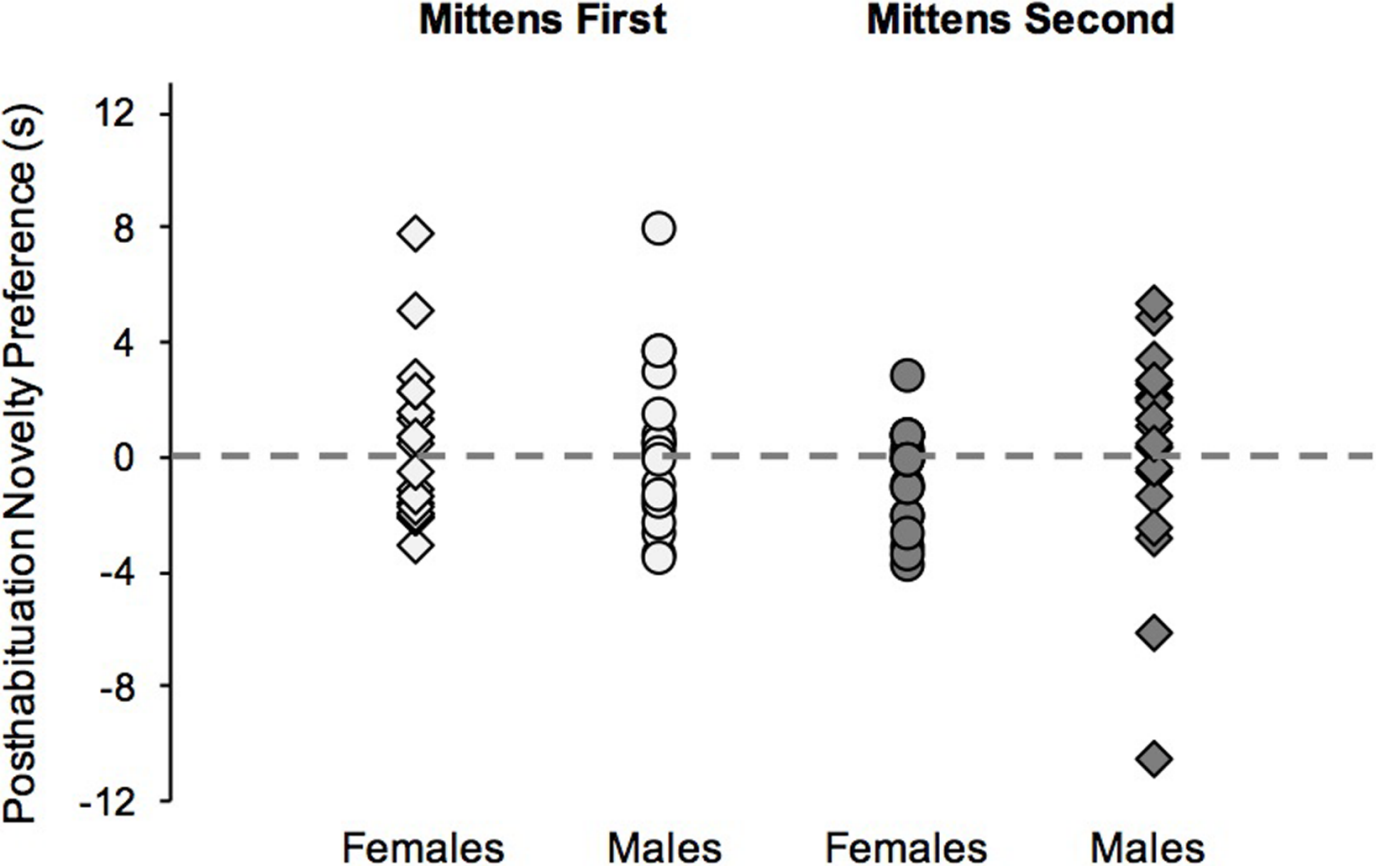
Posthabituation looking times. Posthabituation Novelty Preference scores for individual infants in the Mittens First condition (left two columns) and the Mittens Second condition (right two columns). Data for females and males are plotted separately. The dotted line indicates chance performance.

Fig 3 plots Novelty Preference scores for Mittens First and Mittens Second conditions, separated for females and males. None of the four groups’ scores consistently departed from chance levels (0), *t*s(19) = -1.93 to .50, *ns*, nor did Novelty Preference scores across all 80 infants combined, *t*(79) = -0.07, *ns*; thus these data provide no evidence for MR in any of the groups as a whole (see also Table 1). A 2 (sex) x 2 (condition: Mittens First vs. Mittens Second) univariate ANOVA with Novelty Preference as the dependent variable revealed no significant main effects, and the interaction likewise failed to reach statistical significance, *F*s(1, 76) < 1.0, *ns*. Our first two predictions, therefore, are not supported by these results: Male infants did not show evidence of stronger MR performance than females, and the Mittens First group did not outperform the Mittens Second group.

**Table 1:** Descriptive statistics of novelty preferences and object exploration measures by condition and sex (Mean (SD)), and Across all Infants (Overall).

	Mittens First		Mittens Second			Overall
	Males	Females		Males	Females	
Novelty preference (s)	0.11 (2.73)	0.30 (2.63)		0.24 (3.57)	-0.73 (1.68)	-0.02 (2.71)
Looking only (s)	29.20 (18.80)	20.85 (19.83)		19.81 (12.86)	23.39 (16.46)	23.31 (15.98)
Touching only (s)	99.09 (39.27)	81.50 (35.18)		111.30 (34.44)	99.33 (40.76)	97.80 (38.31)
Visual-Manual Exploration (s)	93.90 (39.70)	124.68 (42.56)		73.23 (36.54)	82.71 (34.84)	93.63 (42.51)
Total Engagement (s)	222.19 (23.81)	227.03 (19.83)		204.34 (35.21)	205.41 (34.52)	214.74 (30.29)

### Object exploration

Infants were fitted with loop Velcro mittens and provided with 4 min exposure to two hook Velcro-covered objects whose configurations and colorings matched the rotating objects seen during habituation and test. All infants readily engaged in the task. We report separate analyses for the two principal dependent measures, Visual-Manual Exploration (coordinated looking and touching) and Total Engagement (coordinated looking and touching plus looking alone plus touching alone). Table 1 provides means and SDs for all object exploration measures: looking at the object without touching, touching the object without looking, and the two principal DVs of interest, Visual-Manual Exploration, and Total Engagement.

A 2 (sex) x 2 (condition) univariate ANOVA with Visual-Manual Exploration as the dependent variable revealed a reliable main effect of sex, *F*(1, 76) = 5.46, *p* = .022, partial *η*^2^ = .067, the result of greater Visual-Manual Exploration on the part of females (*M* = 103.7 s, *SD* = 43.9) relative to males (*M* = 83.6 s, *SD* = 39.1). This effect may stem from emerging hand preferences, as female infants come to use their dominant hand, and perhaps explore objects in coordinated fashion, earlier than do males [59, 60], although sex differences in sticky mittens performance have not been reported previously to our knowledge [48, 55–57, 61]. There was also a reliable main effect of condition, F(1, 76) = 13.22, p = .001, η2 = .148, the result of greater exploration by infants in the Mittens First condition (M = 109.3 s, SD = 43.5) relative to infants in the Mittens Second condition (M = 78.0 s, SD = 35.6), most likely due to fatigue in the latter group from first participating in the MR task. The interaction was not statistically significant.

A 2 (sex) x 2 (condition) univariate ANOVA with Total Engagement as the dependent variable revealed a reliable main effect of condition, *F*(1, 76) = 9.19, *p* = .003, partial *η*^2^ = .108, the result of greater engagement by infants in the Mittens First condition (*M* = 224.6 s, *SD* = 21.8) relative to infants in the Mittens Second condition (*M* = 204.9 s, *SD* = 34.4), again likely stemming from fatigue in the Mittens Second group. The effect of sex and the interaction were not statistically significant.

### Relations between object exploration and MR performance

We used linear regression to examine the association between Visual-Manual Exploration and MR performance (i.e., Novelty Preference), as a function of sex and condition, and their interactions with Visual-Manual Exploration. There were no significant effects, *β*s < 0.20, *p*s > .091.

**Fig 4:**
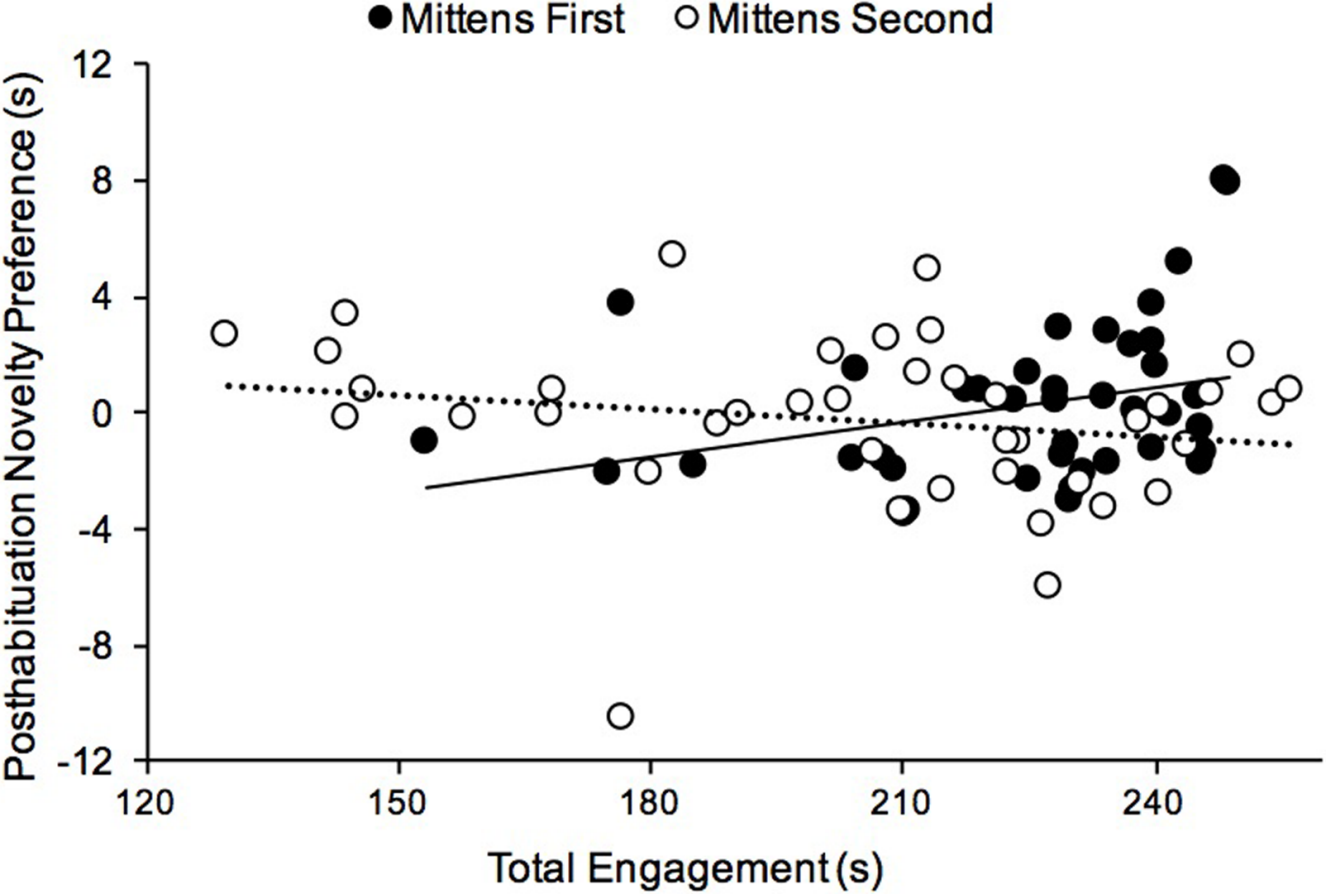
Relations between object exploration and looking times. Total Engagement is plotted against Posthabituation Novelty Preferences for individual infants in the Mittens First and Mittens Second conditions. There was a positive relation between measures for the Mittens First condition (dark symbols, solid trendline) but not for the Mittens Second condition (light symbols, dotted trendline).

Next, we used linear regression to examine the association between Total Engagement and Novelty Preference as a function of sex and condition, and their interactions with Total Engagement. This analysis revealed a significant interaction between condition and Total Engagement, *β* = 0.30, *p* = .018. There were no other reliable effects. The interaction, plotted in Fig 4, stems from differences in relations between Total Engagement and Novelty Preference as a function of condition (task order). Infants in the Mittens First condition who exhibited more Total Engagement with the objects (visual, manual, and the combination) tended to have stronger novelty preferences, Pearson *r* = .31, *p* < .05, in contrast to infants in the Mittens Second condition, *r* = -.19, ns.

## Discussion

The present research investigated 4-month-olds’ mental rotation (MR) performance and one factor that may underlie such performance: object exploration. We hypothesized that variation among individuals in coordinated Visual-Manual Exploration of objects, or in Total Engagement (Visual-Manual Exploration plus looking plus touching), might relate to emerging MR ability in infants. Our results point to similarities in MR performance overall between females and males at 4 months, and a relation between Total Engagement of objects and MR performance. We discuss these findings below.

Previous research suggested that among young infants, only males provide consistent evidence of MR of 2D images, including 2D depictions of 3D objects [30–35]. However, in our sample of 4-month-old infants, and using the same habituation stimuli and looking time methods as prior studies demonstrating MR in 3- and 5-month-old males only [30, 32, 33], we did not find evidence of an overall male advantage (likewise, male and female infants’ performances in this MR task were found to be similar to one another in [62]). Levine and colleagues [15] provided a possible explanation for previous studies’ null results among female infants, and perhaps for the results of the current study. Specifically, the test stimuli employed by Quinn and Liben [34, 35] and Moore and Johnson [32, 33] pictured a mirror-image stimulus object as well as the familiarized stimulus object seen from a novel orientation. It is possible that in these previous studies, female infants recovered attention to both the novel *orientation* of the familiar stimulus object as well as to the novel *structure* of the mirror-image stimulus object, such that no significant novelty or familiarity preference was obtained, despite some ability to mentally rotate and discriminate the familiar stimulus from its mirror image.

We addressed this possibility in the current data set with analyses of female and male infants’ *recovery of interest* (i.e., dishabituation), which measures the extent to which infants look longer at novel test stimuli upon first presentation, and is generally understood to reflect the extent to which infants detect differences between familiar habituation displays and novel test displays [63]. That is, even in the case of males’ and females’ overall null novelty preferences, infants may still have recovered attention to both test stimuli, compared to the familiar habituation stimulus. Recovery of interest was computed by subtracting looking times on the last habituation trial from looking times on the first familiar test trial and first novel test trial. Infants’ attention increased to *both* test stimuli after habituation. For males, *M* recovery to familiar = 2.31 s (*SD* = 4.96), *t*(39) = 2.94, *p* = .005, and *M* recovery to novel = 3.70 s (*SD* = 6.05), *t*(39) = 3.87, *p* < .001. Likewise for females, *M* recovery to familiar = 2.81 s (*SD* = 5.13), *t*(39) = 3.47, *p* = .001, and *M* recovery to novel = 2.06 s (*SD* = 5.92), *t*(39) = 2.20, *p* = .034. Thus, infants in the current study, and possibly female infants in previous studies, appear at minimum to have recognized differences between the habituation stimulus and *both* test stimuli, perhaps noticing and responding to novel surface colors and edge orientations, if not necessarily 3D object structure. These patterns of responding account for the overall null results for MR when novelty preference is taken as the principal dependent measure.

Despite our finding that no group of infants generated Novelty Preference scores that departed significantly from chance, novelty preference remains an important metric of MR in infants. Longer looking at a novel, mirror-image object than at a new view of the familiar habituation object suggests recognition of the familiar object even though it is being seen in a new orientation. Thus, a preference for the novel, mirror-image stimulus is indicative of mental rotation. In the present study, relations between this measure and object exploration revealed positive effects of increased Total Engagement when infants interacted with the object prior to seeing it in the test of MR. Thus, infants who were best able to take advantage of opportunities for visual and manual exploration of complex objects provided better evidence of MR skill when tested immediately afterward. To our knowledge, this is the first study of MR in 4-month-olds. Previously, 3-month-old males and 5-month-old males exhibited significant posthabituation preferences in protocols similar to the test for MR employed in the current study ([33] and [32], respectively). The lack of significant posthabituation preferences among infants as a group in the present study suggests that this ability may be more fragile among infants at this age than previously thought. Accordingly, the infants who demonstrated the strongest novelty preferences in the present study were those whose MR performances were facilitated by their taking most advantage of the opportunity to visually and manually explore objects prior to the MR test. Among much older infants with more robust MR and more developed visual and manual object exploration skills, such relations likely would not be observed.

Interestingly, Visual-Manual Exploration alone did not predict novelty preference as strongly as did Total Engagement. This is somewhat surprising, as visual-manual exploration engages multiple modalities simultaneously, perhaps constituting a particularly informative type of exploration and one that has been linked to object cognition in previous literature (e.g., [45, 64, 65]). Nonetheless, the present study suggests that time spent engaging in synchronous, coordinated visual-manual object exploration contributes to infants’ later MR performances only *in conjunction with* time spent asynchronously exploring the object visually and exploring the object manually. Considering all three of these activities together provided the greatest explanatory power for infants’ later MR performance.

There are good reasons to believe that visual object exploration and manual object exploration need not be simultaneous to contribute to developing object knowledge. For instance, manual-without-visual engagement with an object results in a transformation of that object in space, subsequently providing new visual information about the objects’ configuration when it is later viewed. Moreover, it is largely unclear from previous studies reporting effects of object exploration on MR how much these effects stemmed from manual exploration, visual exploration, or their (synchronous or asynchronous) combination. For instance, Frick and Wang ([38], Experiment 3) examined infants’ MR of an object on a turntable after either hands-on or observational experience with the turntable. In the “hands-on” condition, the infant and their parent could rotate a turntable that was positioned 6 cm away from the infant. In the “other-turning” comparison condition, infants watched an experimenter rotate a turntable that was positioned 62 cm away from the infant. The authors reported that 13- and 14-month-old infants were able to mentally track the orientation of an object on a turntable only after hands-on experience with the turntable carrying another object. However, given the relative distances of the turntables from the infants, the object views provided in the hands-on condition were larger and clearer than the object views provided in the other-turning condition Thus, although the authors framed their finding as an effect of “hands-on” learning, it is possible that their findings had as much to do with these larger, clearer object views as they had to do with the haptic experiences obtained from rotating the turntable. If this is the case, it would not matter if those views were observed synchronously or asynchronously with infants’ hands on the object. Considered in this way, the present findings of a relation between MR and Total Engagement with complex objects—rather than a relationship between MR and coordinated Visual-Manual Exploration alone—might not be particularly surprising.

Importantly, the relation between Total Engagement with objects and MR performances differed by condition (Mittens First versus Mittens Second). If it were simply the case that more advanced infants tend to engage in more self-produced visual and manual object exploration as well as exhibit better mental rotation skill, we should have observed relations between Total Engagement and MR in both conditions. Instead, infants who took greater advantage of the opportunity for object exploration immediately prior to the MR task exhibited larger novelty preferences (consistent with MR ability). This finding suggests that MR ability is affected by Total Engagement with objects over time. This result highlights the importance of embodied experience in the development of object knowledge and suggests that opportunities for self-generated object exploration may be pivotal in promoting both males’ and females’ MR abilities and possibly in developing training programs and early interventions.

More broadly, there could be multiple determinants of developing MR and of the relationship between experiences and MR ability, a possibility in line with a recent study on how self-produced actions relate to children’s spatial skills. Levine and colleagues [11] observed naturally occurring puzzle play during six home visits when children were between 2.2 and 3.8 years of age. When children were 4.5 years of age, they participated in a mental transformation task, and performance was better among children who engaged in greater spontaneous puzzle play during the home visits. Moreover, among the children who played with puzzles, *frequency* of puzzle play was significantly associated with mental transformation performance for both males and females, whereas *quality* of puzzle play (i.e., puzzle difficulty, level of engagement during puzzle play, and spatial language during puzzle play) was significantly associated with mental transformation performance only among females. Such findings, and the results of the current experiment, suggest that particular aspects of visual and hands-on interactions with objects early in development may facilitate the spatial abilities of individual children to different extents.

Additionally, previous research indicates that action experiences, such as playing video games, improve children’s MR skills (e.g., [66, 67]), particularly for those starting with lower spatial skill levels [68]. This might imply that a sample of infants starting with lower spatial skill levels may benefit the most from facilitated hands-on experiences with objects. Future research could use a pre-test/post-test design to investigate this possibility, examining whether infants with the poorest initial MR performances benefit most from experience manipulating objects.

Other possible determinants of MR in infancy have been recently reported. Constantinescu and colleagues [30], for example, found that MR performance (posthabituation novelty preference) in 5-month-old girls (but not boys) was correlated with the stereotypicality of parents’ attitudes about gender, such that more traditional views in parents were associated with poorer performance in female infants; conversely, performance in 5-month-old boys (but not girls) was positively correlated with perinatal testosterone levels. A sex difference in visual object cognition may be due in part to the sexual differentiation of the brain in prenatal and early postnatal life, for instance due to differential pre- and/or peri-natal androgen exposure [69]. Also consistent with this possibility, Lauer and colleagues [31] reported that greater visual interest in male-typed compared to female-typed objects in a preferential looking paradigm was related to better MR performance among male, but not female, 6- to 13-month-olds. Further research is needed to clarify the extent to which relations between object exploration and MR skills result from differential early behavioral experiences and prenatal hormone exposure.

## Conclusions

In summary, the current study provides evidence that MR in infants is facilitated by visual and manual exploration of complex objects when object exploration takes place immediately prior to MR testing. We did not find evidence for sex differences in MR performance overall. Instead, in tandem with recent infant studies of sex differences in relations between MR and parental attitudes [30], MR and androgens [30], and MR and toy preferences [31], our results imply that multiple factors can influence early MR, and may lead to differential trajectories in the development of this spatial skill over time.
